# Tacrolimus exposure during the three-month period following allogeneic stem cell transplantation predicts overall survival

**DOI:** 10.3389/fphar.2025.1517083

**Published:** 2025-04-25

**Authors:** Alzbeta Zavrelova, Katerina Zibridova, Jakub Radocha, Eva Cermakova, Petra Rozsivalova, Pavel Zak, Benjamin Visek, Miriam Lanska, Jana Stevkova, Sara Merdita, Ondrej Slanar, Martin Sima

**Affiliations:** ^1^ 4th Department of Internal Medicine – Hematology, University Hospital Hradec Kralove and Faculty of Medicine in Hradec Kralove, Charles University, Hradec Kralove, Czechia; ^2^ Department of Medical Biophysics, Faculty of Medicine in Hradec Kralove, Charles University, Hradec Kralove, Czechia; ^3^ Department of Clinical Pharmacy, Hospital Pharmacy, University Hospital Hradec Kralove and Faculty of Pharmacy in Hradec Kralove, Charles University, Hradec Kralove, Czechia; ^4^ Institute of Pharmacology, First Faculty of Medicine, Charles University and General University Hospital in Prague, Prague, Czechia

**Keywords:** allogeneic stem cells, transplantation, tacrolimus, pharmacokinetics, nonlinear mixed-effects model

## Abstract

**Objectives:**

The objective of this study was to investigate the relationship between both short-term and long-term tacrolimus exposure and overall survival after allogeneic stem cell transplantation and to propose individualized tacrolimus dosing based on the population pharmacokinetic model.

**Study design:**

Tacrolimus exposure during the first 3 months of therapy after transplantation was calculated using therapeutic drug monitoring data from all patients who underwent allogeneic stem cell transplantation from 2016 to 2018. The optimal upper level was determined using ROC analysis, and the impact of cutoff tacrolimus exposure values on overall survival of patients was assessed together with other transplant variables using multivariate analysis. The population pharmacokinetic model was developed using a nonlinear mixed-effects modeling method, and the optimal tacrolimus dose was proposed.

**Results:**

A total of 86 patients were included in the outcome analyses. Except for the disease risk category, age ≥55 years, and female-to-male donor, tacrolimus exposures of the area under the curve of trough concentrations (AUC_tc_) ≥ 222 ng h/mL, ≥258 ng h/mL, and ≥160 ng h/mL during the whole three-month period, second month, and third month of therapy, respectively, were also found to be statistically significant for overall survival in univariate analysis. These AUC_tc_ values were independent variables for overall survival in multivariate analysis, with RR of 3.01 (P = 0.0056), 3.22 (P = 0.0058), and 2.93 (P = 0.0184) for the whole three-month period, second month, and third month of therapy, respectively. The disease risk category (RR 7.11; P < 0.0001), age (RR 2.45; P = 0.0214), and non-myeloablative conditioning (RR 3.39; P = 0.0014) were also significant factors influencing survival in multivariate analysis. Tacrolimus volume of distribution was 127.1 L and was not affected by any of the tested covariates, whereas clearance decreased with age according to the equation $\text{CL}=7.94\times e^{-0.0085\times \text{age}}$ and was reduced by 23% in patients who underwent repeat transplantation.

**Conclusion:**

Except for the disease risk category, age, and non-myeloablative conditioning, exposure to tacrolimus is an independent predictor of overall survival and should not exceed trough levels of 10.7 ng/mL during the second month and 6.8 ng/mL during third month after transplantation. In order to reach this target, nomogram for estimation of the maximal initial tacrolimus daily dose was developed based on the population pharmacokinetic model.

## 1 Introduction

Allogeneic hematopoietic transplantation is frequently the only curative standard therapeutic method for many hematological diseases. For a successful outcome after transplantation, it is essential to use immunosuppression, usually for a limited period after the procedure, to allow new immunocompetent cells to develop immune tolerance and to prevent the onset of graft-versus-host disease (GVHD). A calcineurin inhibitor, in combination with low-dose methotrexate or mycophenolate mofetil (MMP), is used in the majority of European centers, and usually, T-cell depletion is performed (The EBMT Handbook, 2024). Cyclosporine and tacrolimus, as the two calcineurin inhibitors, are accepted and are practically interchangeable in this indication (Nash et al., 2005; Ratanatharathorn et al., 1998).

Tacrolimus is increasingly being used over cyclosporine, largely due to its more predictable pharmacokinetics (Ratanatharathorn et al., 1998; Nash et al., 2000). However, it has a narrow therapeutic window, and monitoring of whole blood concentrations is necessary after transplantation. Despite its widespread use, the recommended blood level for patients after allogeneic hematopoietic transplantation is not precisely defined as the target concentration has been extrapolated from solid organ transplantations, with the range of 5–20 ng/mL (Torlen et al., 2016; Mori et al., 2012; Sharma et al., 2021). During the first year after transplantation, immune tolerance usually develops, with immune reconstitution through the thymic pathway, as opposed to solid organ transplantation. Therefore, the immunosuppressive strategy is quite different from solid organ transplantation (van den Brink et al., 2015).

Therapeutic blood levels of calcineurin inhibitors are maintained until the start of drug withdrawal, usually at approximately day 100 after transplantation (Sharma et al., 2021; Ganetsky et al., 2016). Some studies suggest the possibility of earlier dose reduction, which may lead to better disease control, especially in high-risk disease (Abraham et al., 1997; Schmid et al., 2006; Offer et al., 2015). The association between low levels of calcineurin inhibitors and the occurrence of acute graft-versus-host disease (aGVHD) in the early phase (first month) after transplantation is widely described (Mori et al., 2012; Sharma et al., 2021; Ganetsky et al., 2016; Offer et al., 2015; Hagen et al., 2019; Malard et al., 2010; Bianchi et al., 2019). However, only a single study describes prolonged tacrolimus monitoring (up to 6 weeks) and the association between its low levels, that is, levels below 10 ng/mL, and aGVHD (Tian et al., 2019). Here, aGVHD is associated with low levels of calcineurin inhibitors, and optimal values from these studies seem to be above 10 ng/mL. On the other hand, high calcineurin inhibitor levels may lead to inappropriate immunosuppression that may cause disease relapse or opportunistic infections; however, few studies associate higher levels of calcineurin inhibitors with lower overall survival or risk of relapse (Wong et al., 2005; Craddock et al., 2010; Bacigalupo et al., 1991; Braidotti et al., 2024). The greatest mortality after transplantation for malignant diseases is caused by relapse of the disease, which is thus the most important predictor for overall survival (Schmalter et al., 2024). Again, these studies only describe a short period of drug levels monitoring after stem cell transplantation, with a maximum of 21 days, as described by Craddock et al. (2010). Therefore, it is not possible to reliably extrapolate optimal therapeutic calcineurin inhibitor levels in the later phase following transplantation needed to achieve the best clinical outcomes.

The primary aim of this study was to investigate the relationship between both short-term and long-term tacrolimus exposure and overall survival in the context of other clinical covariates possibly affecting patients’ overall survival after allogeneic stem cell transplantation. The secondary objective was to develop a tacrolimus population pharmacokinetic model in order to propose model-based individualization of tacrolimus dosing.

## 2 Methods

### 2.1 Study design and data acquisition

This study was approved by the local Ethics Committee of the University Hospital Hradec Kralove under the registration number 202409P01 on 19 September 2024, and was conducted according to the Declaration of Helsinki. Due to the retrospective nature of this study, which involved only the analysis of routine clinical data, study-specific informed consent was waived. The collection and processing of anonymized data is in the public interest.

The study was designed as an open-label retrospective observational analysis in all adult patients (age ≥18 years) who underwent allogeneic stem cell transplantation in the 4^th^ Department of Internal Medicine–Hematology, University Hospital, and the Faculty of Medicine in Hradec Kralove, Charles University, in 2016–2018 and who were treated with tacrolimus and had measured tacrolimus whole blood concentrations as a part of routine therapeutic drug monitoring (TDM). Due to its binding to erythrocytes, tacrolimus was measured in whole blood (EDTA vacuum blood collection tube). Whole blood drug concentration was measured using a validated method of the chemiluminescent microparticle immunoassay (CMIA) on the ARCHITECT i1000SR (Abbott Diagnostics, Czech Republic).

For a population pharmacokinetic analysis, patients who underwent both first and repeated allogeneic stem cell transplantation were evaluated (to assess the impact of repeat transplantation as a potential covariate of tacrolimus pharmacokinetics), whereas for statistical analysis of factors influencing the overall survival, only patients who underwent first transplantation were evaluated (to prevent result distortion due to the possible re-evaluation of the same patients).

Patients’ diseases were categorized as high or low risk according to disease characteristics. Patients with acute myeloid leukemia (AML) or acute lymphoblastic leukemia (ALL) who were not in remission, had adverse cytogenetics beyond the first complete remission (CR), had myelodysplastic syndrome (MDS) with a blast count ≥5%, or had adverse cytogenetics, chronic lymphoblastic leukemia (CLL), and lymphoma not in remission at the time of transplantation were considered high risk, whereas the remaining patients were classified as low risk. The disease risk category was used to accommodate the heterogeneity of diseases in our cohort. Analyses were performed to assess a variety of peri-transplant and transplant variables that were retrieved from the hospital information system. All HLA typing was done by using a high-resolution technique, with 10/10 alleles.

Tacrolimus treatment was initiated at a dose of 0.03 mg/kg by continuous infusion on the day before transplantation and switched to oral administration around day 11 after transplantation. The dose was adjusted if concomitant voriconazole or posaconazole was used. Tacrolimus levels were measured twice weekly during hospitalization and subsequently once weekly during outpatient visits with a target range of 5–15 ng/mL. MMP treatment started 4 h after completion of stem cell infusion with a dose of 15 mg/kg twice daily. Tapering of immunosuppression was done from day 90 for tacrolimus, and MMP was rapidly withdrawn around day 56.

### 2.2 Statistical analysis

For univariate and multivariate analyses, tacrolimus exposure was calculated using the trapezoidal method as the area under the curve of trough concentrations (AUC_tc_) during the period of first, second, and third months of tacrolimus medication and during the whole three-month period, normalized per 24 h.

The cutoff value most significant for survival was calculated using receiver operating characteristic (ROC) curves. These values were then used for further analyses.

In univariate analysis, clinical factors and AUC_tc_ values for the whole period of 3 months and for the first, second, and third months were analyzed as separate factors in order to further explore the cutoff values for each treatment period. For this reason, multivariate analysis was also performed for each period. However, the whole three-month period was considered a relevant independent analysis for exploring the effect of clinical factors on the overall survival.

Univariate Cox regression was used to find potential factors predicting the overall survival. Then, multivariate Cox regression was calculated. The level of statistical significance was set at α = 0.05. All analyses were performed using NCSS 2023 Statistical Software (NCSS, LLC. Kaysville, Utah, United States, ncss.com/software/ncss).

### 2.3 Pharmacokinetic population model

As only trough levels of tacrolimus are routinely collected during TDM, it is not possible to reliably describe the absorption phase of the pharmacokinetic profile using this sampling. Therefore, we developed a population pharmacokinetic model based only on concentrations collected during initial intravenous therapy, where the absorption phase is not applicable but the distribution and elimination phases can be reliably described.

Whole blood concentration–time profiles of tacrolimus were analyzed using the nonlinear mixed-effects modeling method. The model parameters were assumed to be log-normally distributed and were estimated by maximum likelihood using the stochastic approximation expectation maximization (SAEM) algorithm within Monolix Suite software, version 2021R2 (Lixoft SAS, Antony, France).

For the structural model, one- and two-compartment models with first-order and Michaelis–Menten elimination kinetics were tested. Log-normally distributed interindividual variability terms with estimated variance were tested on each pharmacokinetic parameter. Constant, proportional, and combined error models were tested for the residual error model. The most appropriate model was selected based on the objective function value (OFV), Akaike information criterion (AIC) and Bayesian information criterion (BIC) differences, adequacy of the goodness-of-fit (GOF) plots, and low relative standard errors (RSEs) of the estimated PK parameters.

Age, body weight, height, body mass index, body surface area, and serum creatinine were tested as continuous covariates, whereas gender, diagnosis, first or repeated transplantation, achievement of remission prior to the transplantation, cytomegalovirus reactivation, preparative regimen, sequential chemotherapy, and co-medication with azole antifungals were tested as categorical covariates of pharmacokinetic parameters. Co-medication was evaluated in a dose-independent manner (Sima et al., 2015). A preliminary graphical assessment and univariate association using Pearson’s correlation test of the effects of covariates on estimated pharmacokinetic parameters was conducted. The covariates with P < 0.05 were considered for the covariate model. Afterward, a stepwise covariate modeling procedure was performed. For the model selection, forward addition and backward elimination methods were used. In the forward addition method, a decrease in OFV of more than 3.84 points between nested models (P < 0.05) was considered statistically significant, assuming a χ^2^-distribution. In the backward elimination method, covariates were retained in the model if difference in OFV was greater than 6.64 points between nested models (P < 0.01). Additional criteria for model selection were reasonably low RSE values of the estimates of model parameters, physiological plausibility of the obtained parameter values and the covariates found, and the absence of bias in GOF plots.

Model adequacy was evaluated using GOF plots. Observed concentrations were plotted against individual and population predictions, whereas the normalized prediction distribution errors (NPDEs) were plotted against the time and population predictions. To evaluate the stability of the model, a bootstrap analysis was performed. In this procedure, 250 replicates of the original data were generated, and the parameter estimates for each of the 250 samples were re-estimated using R package Rsmlx for Monolix Suite (Lixoft SAS, Antony, France) in the final model. The median and 95% confidence intervals (CI) obtained for each of the parameters estimated for bootstrap samples were compared with the estimates in the final model.

## 3 Results

### 3.1 Patients and treatment characteristics

Allogeneic stem cell transplantation was performed in 100 patients during 2016–2018. A total of 86 patients underwent first transplantation and were evaluated for clinical outcomes. Detailed characteristics of the patients are summarized in Table 1.

**TABLE 1:** Patient and treatment characteristics.

Characteristic	n (%)/median (range)
Demographics	
Sex (female/male)	36 (41.9)/50 (58.1)
Age at STC	58 (20–72)
Body weight (kg)	82 (49–133)
Height (cm)	172.5 (152–200)
BMI (kg/m^2^)	26.0 (16.8–42.7)
BSA (m^2^)	1.96 (1.49–2.49)
≥55 years	31 (36.0)
Diagnosis	
AML	43 (50.0)
MDS	12 (14.0)
ALL	8 (9.3)
PCL + MM	3 (3.5)
CLL + lymphoma	11 (12.8)
Histiocytosis	1 (1.2)
HLH	1 (1.2)
MPL	7 (19.8)
Risk	
Low	35 (40.7)
High	51 (59.3)
Stem cell type	
PBSC	86 (100)
Donor type	
MRD	29 (33.7)
MUD	42 (48.8)
MMUD (9/10 match)	15 (17.4)
Female-to-male donor	12 (14.0)
CMV status	
CMV P+/D-	30 (34.9)
Other	56 (65.1)
Conditioning	
Myeloablative	51 (59.3)
Non-myeloablative	35 (40.7)
Thymoglobulin 6 mg/kg	85 (98.8)
ATG 60 mg/kg	1 (1.2)
GVHD	
aGVHD	23 (26.7)
aGVHD 2–4	17 (19.8)
chGVHD	13 (15.1)
chGVHD moderate to severe	8 (9.3)

AML, acute myeloid leukemia; ALL, acute lymphoblastic leukemia; MDS, myelodysplastic syndrome; PCL, plasma cell leukemia; MM, multiple myeloma; CLL, chronic lymphocytic leukemia; HLH, hemophagocytic lymphohistiocytosis; MPL, myeloproliferative disease; PBSC, peripheral blood stem cell; MRD, matched related donor; MUD, matched unrelated donor; MMUD, mismatched unrelated donor; CMV, cytomegalovirus; ATG, anti-thymocytic globulin (Grafalon); GVHD, graft-versus-host disease.

Most patients were transplanted for malignant disease, except one, who received transplantation for congenital hemophagocytic lymphohistiocytosis (HLH) with severe neurological complications. Conditioning was based on busulfan in 72 patients who received intravenous busulfan at doses of 6.4–13.2 mg/kg. Total body irradiation (TBI) of 12 Gy was used in 10 patients, mainly with ALL. Two patients received TBI of only 2 Gy: one patient received the treosulfan-based regimen (36 mg/kg) and one patient with plasmocellular leukemia received the fludarabine/melphalan regimen. Of 43 AML patients, 16 were not in remission before transplantation, and of 12 patients with MDS, 10 had ≥5% blasts in bone marrow. Adverse cytogenetics was present in 11 AML patients and six MDS patients. Immunosuppression consisted of tacrolimus, mycophenolate mofetil, and rabbit anti-thymocyte globulin (Thymoglobulin) 3 mg/kg on days −2 and −1 in the whole study population, except one patient with HLH who received rabbit anti-human T-lymphocyte Ig (Grafalon) 60 mg/kg instead of Thymoglobulin. According to the established treatment regimen, all patients were co-treated with MMP and azole antifungals (28 patients received voriconazole, 20 patients received posaconazole, and 52 patients were treated with fluconazole).

### 3.2 ROC—identification of cutoff values for tacrolimus exposure and age

ROC curves were used to identify the most appropriate upper cutoff values for tacrolimus exposure over time and the effect of age on the clinical outcome (Supplementary Data). The maximum AUC_tc_ values were ≥222 ng h/mL, ≥279 ng h/mL, ≥258 ng h/mL, and ≥160 ng h/mL for the whole period, and first, second, and third months, respectively. We were not able to identify a lower cutoff value optimal for GVHD control, as low levels of tacrolimus were scarce in our dataset.

Overall survival was independently affected by age, with patients ≥55 years having a worse outcome.

### 3.3 Survival and univariate analysis

**FIGURE 1:**
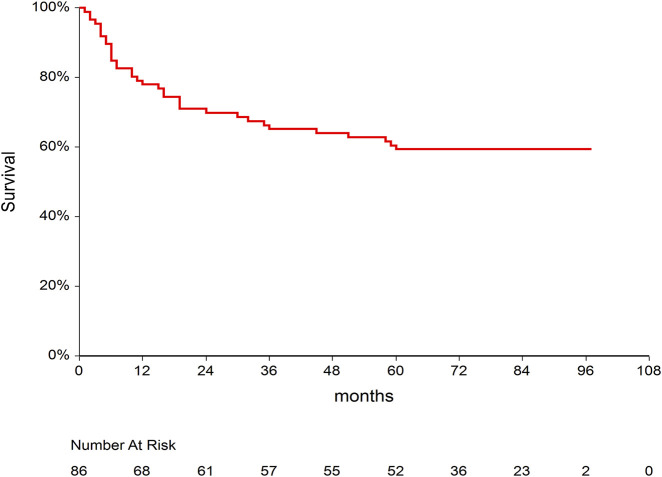
Kaplan–Meier curve for overall survival after allogeneic stem cell transplantation in our study population.

At the time of analysis, 35 (40.7%) of 86 included patients died with a median (95% CI) follow-up time of 11 months (Mori et al., 2012; Sharma et al., 2021; van den Brink et al., 2015; Ganetsky et al., 2016; Abraham et al., 1997; Schmid et al., 2006; Offer et al., 2015; Hagen et al., 2019; Malard et al., 2010; Bianchi et al., 2019; Tian et al., 2019). Median survival time of the whole cohort was not reached (Figure 1). Thirty-one (36%) out of 86 patients experienced disease relapse. The causes of death were relapse in 22 cases, infection in five cases, GVHD in three cases, complications after second allogeneic transplantation, which was performed because of relapse, in three patients, secondary malignancy in one patient, and sudden death in one patient in remission.

Univariate analysis of the whole group is summarized in Table 2. The disease risk category proved to be the most decisive factor influencing survival. Age and female-to-male donor were further significant factors for overall survival. Interestingly, HLA mismatch, non-myeloablative conditioning, aGVHD, CMV high-risk group (donor-negative and patient-positive), and relative donor status did not reach statistical significance in univariate analysis. We also found that AUC_tc_ values for the whole period of 3 months and also separately for the second and third months of therapy were significant factors affecting overall survival.

**TABLE 2:** Univariate analysis of factors predicting overall survival.

	RR	95% CI	P-value
Disease risk	**4.58**	**1.90–11.06**	**0.0001**
Female-to-male donor	**2.71**	**1.26–5.83**	**0.0191**
Age ≥ 55 years	**2.13**	**0.97–4.70**	**0.0455**
CMV D-/P+	2.00	0.91–4.41	0.0686
Matched related donor	1.84	0.95–3.58	0.0771
Conditioning, non-myeloablative	1.75	0.90–3.40	0.0987
HLA mismatch	0.96	0.40–2.31	0.9215
aGVHD	0.95	0.44–2.02	0.8888
AUC_tc__1st month ≥279 ng h/mL	1.96	0.96–4.01	0.0556
AUC_tc__2nd month ≥ 258 ng h/mL	**2.24**	**1.10–4.56**	**0.0256**
AUC_tc__3rd month ≥ 160 ng h/mL	**3.34**	**1.44–7.77**	**0.0022**
AUC_tc__whole period ≥ 222 ng h/mL	**2.94**	**1.44–6.03**	**0.0020**

Bold value represents statistically significant factors.

### 3.4 Survival and multivariate analysis

First, we analyzed the AUC_tc_ for the whole study period of 3 months altogether. In the Cox RH model, overexposure above the tacrolimus AUC_tc_ cutoff limit (Table 3A) was an independent factor for decreased overall survival, along with other risks (risk category, age, and non-myeloablative conditioning).

**TABLE 3:** Multivariate analysis of factors predicting overall survival.

(A) whole 3-month period	RR	95% CI	P-value
Age ≥55 years	**2.45**	**1.09–5.53**	**0.0214**
Disease risk	**7.11**	**2.64–19.13**	**<0.0001**
Conditioning non-myeloablative	**3.39**	**1.60–7.20**	**0.0014**
HLA mismatch	2.88	0.97–8.52	0.0674
CMV D-/P+	1.71	0.75–3.90	0.1886
Female-to-male donor	1.87	0.73–4.80	0.1991
Matched related donor	1.35	0.60–3.06	0.4738
aGVHD	1.08	0.47–2.49	0.8547
AUC_tc_ ≥222 ng h/mL	**3.01**	**1.34–6.76**	**0.0056**

(B) first-month period	RR	95% CI	P-value
Age ≥55 years	**2.31**	**1.01–5.27**	**0.0357**
Disease risk	**7.53**	**2.86–19.86**	**<0.0001**
Conditioning non-myeloablative	**3.23**	**1.49–6.99**	**0.0028**
HLA mismatch	2.09	0.76–5.75	0.1730
CMV D-/P+	1.49	0.64–3.47	0.3476
Female-to-male donor	2.30	0.88–6.04	0.0959
Matched related donor	1.42	0.64–3.12	0.3920
aGVHD	1.19	0.51–2.77	0.6908
AUC_tc_ ≥279 ng h/mL	1.89	0.86–4.14	0.1069

(C) second-month period	RR	95% CI	P-value
Age ≥55 years	1.95	0.81–4.69	0.1207
Disease risk	**9.82**	**3.44–27.98**	**<0.0001**
Conditioning non-myeloablative	**4.21**	**1.83–9.72**	**0.0007**
HLA mismatch	1.67	0.56–5.04	0.3757
CMV D-/P+	**2.57**	**1.03–6.40**	**0.0322**
Female-to-male donor	1.46	0.55–3.88	0.4560
Matched related donor	1.71	0.75–3.91	0.2096
aGVHD	1.07	0.44–2.60	0.8874
AUC_tc_ ≥258 ng h/mL	**3.22**	**1.41–7.34**	**0.0058**

(D) third-month period	RR	95% CI	P-value
Age ≥55 years	**2.45**	**1.03–5.84**	**0.0312**
Disease risk	**7.05**	**2.52–19.70**	**<0.0001**
Conditioning non-myeloablative	**3.43**	**1.49–7.88**	**0.0034**
HLA mismatch	1.85	0.62–5.57	0.2903
CMV D-/P+	1.43	0.58–3.53	0.4286
Female-to-male donor	1.72	0.64–4.60	0.2875
Matched related donor	1.30	0.55–3.05	0.5503
aGVHD	1.09	0.44–2.68	0.8594
AUC_tc_ ≥160 ng h/mL	**2.93**	**1.15–7.44**	**0.0184**

Bold value represents statistically significant factors.

Then, we further analyzed the three monthly tacrolimus exposures separately. We hypothesized that the desired tacrolimus exposures for the first, second, and third months of therapy differ as the immune tolerance evolves gradually after transplantation. Our analysis has identified optimal upper cutoff values for tacrolimus AUC_tc_ predicting better overall survival for the second and third months. The respective value for the first month was not statistically significant in multivariate analysis (Table 3B–D).

### 3.5 Pharmacokinetic population model

In addition to the above 86 patients undergoing their first allogeneic transplantation, the pharmacokinetic analysis included tacrolimus concentration data from an additional 14 patients undergoing repeated transplantation. Therefore, a total of 349 tacrolimus whole blood concentrations from 100 transplantations (two to seven concentrations per case) were included in the analysis.

A one-compartment model with linear elimination kinetics best fitted tacrolimus concentration–time data. A combined error model was the most accurate for the description of residual and interpatient variability. The pharmacokinetic model was parametrized in terms of volume of distribution (Vd) and clearance (CL). Among all the tested variables, the addition of age and repeated transplantation as covariates of tacrolimus CL was shown to be the most appropriate for improving the model, whereas tacrolimus Vd did not correlate with any of the tested variables.

The population pharmacokinetic estimates and bootstrap results in the final population model are summarized in Table 4.

**TABLE 4:** Estimates of the final tacrolimus population pharmacokinetic model and bootstrap results based on 250 simulations.

	Final model		Bootstrap analysis
Parameter	Estimate	RSE (%)	Median (95% CI)
Fixed effect			
Vd_pop (L)	127.1	7.9	124.9 (123.6–126.6)
CL_pop (L/h)	7.94	15.3	7.70 (7.58–7.94)
β_CL_age (years)	−0.0085	32.2	−0.0081 (−0.0085–0.0076)
β_CL_repeated Tx (if yes)	−0.26	37.2	−0.23 (−0.25–0.21)
Standard deviation of the random effects			
Ω_Vd	0.45	18.6	0.43 (0.41–0.44)
Ω_CL	0.29	8.6	0.28 (0.28–0.29)
Error model parameter			
Constant	0.0018	15.6	0.0018 (0.0018–0.0019)
Proportional	0.17	11.2	0.17 (0.17–0.18)

pop represents the typical value of the parameter; β represents the covariate effect on the parameter; Vd is the volume of distribution; CL is the clearance.

The final equations describing the relationships between tacrolimus pharmacokinetic parameters and their covariates are the following:

$$\text{Vd}={\text{Vd}}_{\text{pop}},$$

$$\text{CL}={\text{CL}}_{\text{pop}}\times e^{\beta_{{\text{CL}}_{\text{age}}}\times \text{age}}\times e^{\beta_{{\text{CL}}_{\text{repeated}}\text{Tx}}\;\left(\text{if}\;\text{yes}\right)},$$

where pop represents the typical value of the parameter and β represents the covariate effect on the parameter.

Therefore, the typical value of tacrolimus volume of distribution in our study population was 127.1 L and was not affected by any of the tested covariate. Clearance can be estimated based on the age of the patient and whether he/she is undergoing a first or repeat transplantation using the following equation:

$$\text{CL}=7.94\times e^{-0.0085\times \text{age}}\times e^{-0.26}\left(\text{if repeated Tx}\right)\;\frac{L}{h}.$$

For example, in a 57-year-old patient (the median age in our population), who undergoes his/her first transplantation, the estimated tacrolimus clearance is 4.89 L/h. With repeated transplantation, the clearance of tacrolimus is reduced by 23% (e^−0.26^ = 0.77), so the estimated tacrolimus clearance for the same patient in the second transplantation would be 3.77 L/h.

The diagnostic GOF plots for the final covariate model did not indicate major deviations. As shown in Table 4, the RSE (maximum 37.2%) revealed that all pharmacokinetic parameters in the model were precisely estimated. All median parameter values in the bootstrap procedure were consistent with the values obtained in the final model fit (maximum difference of 13%), indicating the reliability of the parameter and the random-effect estimates.

## 4 Discussion

The period during which tacrolimus levels were analyzed in this study and related to survival and clinical data is exceptionally long. Most previous analyses of tacrolimus levels aimed to prove a connection between the drug levels and the occurrence of aGVHD after an allogeneic stem cell transplant. These studies showed similar results for the minimum tacrolimus whole blood trough concentration needed to avoid an increased risk of aGVHD. In the study by Hagen et al., the optimal trough levels of tacrolimus were 10–15 ng/mL for the first 4 weeks (aGVHD grade II–IV was present in 26.5% of patients), and according to the study by Sharma el al., tacrolimus levels should be ≥10.15 ng/mL during the first week (aGVHD grade II–IV was present in 40% of patients) for lower risk of aGVHD (Sharma et al., 2021; Hagen et al., 2019). Ganetsky el al. reported that first-week levels should be >12 ng/mL (aGVHD grade II–IV of 42.8%), and Offer et al. found that in children, levels should be above 10 ng/mL during the third week (aGVHD grade II–IV 33%) (Ganetsky et al., 2016; Offer et al., 2015). Yoshikawa et al. suggested that prolonged exposure to tacrolimus blood levels ≥10 ng/mL early post-transplantation may avert the severity of acute graft-versus-host disease (Yoshikawa et al., 2025). We were not able to find a connection between tacrolimus exposure and the risk of aGVHD. First, the incidence of aGVHD grade II–IV was low (19.8%) in our study, likely due to the uniform use of T-cell depletion in all patients, as opposed to the study by Ganetsky, where no antithymocyte globulin was used, and to the study by Sharma et al. and Offer et al., where only 62.7% and 56.7% received immunotherapy with antithymocyte globulin or alemtuzumab (Sharma et al., 2021; Ganetsky et al., 2016; Offer et al., 2015). Second, tacrolimus trough levels during the first month after transplant in our population mostly exceeded the previously reported minimum tacrolimus trough values. The median (Q1–Q3) tacrolimus trough values by days 10 and 28 were 10 ng/mL and 11.87 (9.2–13.7) ng/mL, respectively (van den Brink et al., 2015; Ganetsky et al., 2016; Abraham et al., 1997; Schmid et al., 2006; Offer et al., 2015).

Our study did not demonstrate a statistically significant effect of tacrolimus exposure/levels during the first month on the overall survival in the multivariate model. In contrast, Wong at al. found that tacrolimus exposure during the first 11 days correlated with survival and described the optimal tacrolimus trough range of 7–9 ng/mL (Wong et al., 2005). In the abovementioned study, Sharma et al. reported that tacrolimus trough levels ≥11.55 ng/mL during the second week correlated with relapse incidence (Sharma et al., 2021). However, in both of these studies, the authors followed only a very short period of time after transplantation, and therefore, nothing can be inferred from these studies about tacrolimus exposure during the entire treatment period. In our cohort, it appeared that the longer period we analyzed, the higher level of statistical significance we achieved. Therefore, in the case of short time period of analysis, the results may be inaccurate as they fail to capture tacrolimus exposure over time.

A limitation of our study is that due to the very stable and predictable pharmacokinetics of tacrolimus and well-performed routine dose-adjusted TDM, there were only a minimum number of patients in our cohort who did not achieve minimal therapeutic drug exposure (Venkataramanan et al., 1995). Therefore, we were not able to identify minimum tacrolimus exposures/levels predictive of clinical outcomes (Venkataramanan et al., 1995).

On the other hand, using ROC analysis, we identified an upper limit for each of the 3 months that strongly correlated with the overall survival in the case of months 2 and 3, as well as for the entire three-month period.

Maximum tacrolimus exposure limits decreased over time since transplantation, suggesting that tacrolimus levels should be adjusted/reduced to achieve the best outcome of allogeneic transplantation during the approximately three-month therapeutic period of tacrolimus administration. During the first, second, and third months, the tacrolimus exposure, expressed as AUC_tc_, should not exceed 279 ng h/mL (corresponding to a trough concentration of 11.6 ng/mL), 258 ng h/mL (corresponding to a trough concentration of 10.8 ng/mL), and 160 ng h/mL (corresponding to a trough concentration of 6.7 ng/mL), respectively. This principle of decremental exposure over time is consistent with previously published studies, where early tacrolimus dose reduction was associated with very good clinical outcomes (Abraham et al., 1997; Schmid et al., 2006). In fact, adjusting higher blood levels allows for earlier tapering of immunosuppression.

This further highlights the need to monitor tacrolimus levels even in the later periods after transplantation, when disproportionately high exposure and resulting immunosuppression negatively affect the prognosis of patients after allogeneic stem cell transplantation.

Although our study was not designed to directly compare the importance of TDM at different time points after transplantation, when integrating the available data from previous studies with our results, we conclude that in the first month after transplantation, it is probably more important to achieve a minimum therapeutic level to prevent aGVHD, and later, it may be more important not to overexpose tacrolimus/over-suppress the immune system, which increases the risk of relapse and adverse survival outcomes.

There are no substantial discrepancies between univariate and multivariate analyses. Although the non-myeloablative conditioning was not a significant parameter for survival in the univariate analysis, it later turned out as a significant covariate in the multivariate analysis. This apparent discrepancy is probably due to an imbalance between patients receiving ablative and non-myeloablative regimens, as non-myeloablative conditioning is used for older and frail patients, whereas most of younger patients treated for malignancy receive ablative regimens. Thus, the worse survival of older patients in univariate analysis likely overshadowed the less significant effect of non-myeloablative conditioning. Therefore, non-myeloablative conditioning only emerged as a significant covariate after the correct adjustment for age in the multivariate analysis.

We developed a population pharmacokinetic model for tacrolimus, in which the typical value of volume of distribution was 127 L and was not affected by any of the covariates tested, whereas clearance decreased with age according to the following equation: $\text{CL}=7.94\times e^{-0.0085\times \text{age}}$ . Clearance was further reduced by 23% in patients who underwent a repeat transplantation. Our observation that tacrolimus clearance decreases with age seems physiologically plausible. Tacrolimus is completely metabolized before its elimination from the organism, mainly via CYP3A enzymes (Venkataramanan et al., 1995). Although this subfamily of enzymes possesses relatively few age-dependent changes in its functional capacity, some reports indicate a decrease in the hepatic CYP3A content and the expression pattern in the elderly (Konstandi and Johnson, 2023).

As tacrolimus is a substrate of CYP3A enzymes, exposure to tacrolimus may also be altered by polymorphisms in these enzymes. Although a rather minor contribution of the CYP3A genotype to intravenous tacrolimus exposure was observed in patients after allogeneic stem cell transplantation (Pasternak et al., 2022), another study in a renal transplant population reported that *CYP3A5* expressors required approximately twice the dose of tacrolimus to maintain comparable exposure compared to non-expressors, and the frequency for CYP3A5 expressors was significantly higher in blacks (100%) and mixed-ancestry patients (76%) than in the white population (12%) (Muller et al., 2020). Due to the fact that CYP3A genotyping is not routinely performed prior to tacrolimus administration and that our study was performed in a retrospective design, we could not examine this marker as a covariate in our model. It should be noted that there were only white patients in our study population. In addition, possible inclusion of the genotype in the model would limit its practical utility, as most clinical sites would also not have the genotype available. Nevertheless, it should be acknowledged that the CYP3A genotype may be a source of undescribed variability in the pharmacokinetics of tacrolimus in the population, particularly where there is a higher proportion of black people.

Our expectation that co-administration of azole antifungals (as known inhibitors of the CYP3A family) would result in a drug–drug interaction characterized by increased exposure to tacrolimus (as a substrate of CYP3A) was not detected. Voriconazole and posaconazole have been reported to be potent CYP3A inhibitors, and their co-administration can lead to up to 5-fold increase in tacrolimus dose-corrected trough concentrations (Vanhove et al., 2017). However, the CYP3A genotype and several clinical variables, such as age and hematocrit, have been identified as factors that blunt this interaction. However, the main reason why this interaction did not prove significant in our analysis is probably the fact that all patients were treated with azole antifungals. In total, 28 patients received voriconazole, 20 patients received posaconazole, and 52 patients were treated with fluconazole, which is considered a moderate inhibitor of CYP3A (Gaies et al., 2011). There was a trend toward lower tacrolimus clearance in voriconazole and posaconazole subgroups than in the fluconazole subgroup, with a median (min–max) tacrolimus clearance in voriconazole, posaconazole, and fluconazole subgroups of 4.50 (1.58–10.70), 4.77 (2.74–8.93), and 5.12 (2.69–8.50) L/h, respectively. Therefore, small differences in tacrolimus clearance among patient subgroups who had a concomitant azole agent and were thus exposed to the drug–drug interaction, combined with the relatively high variability (which may be caused, i.e., by the CYP3A genotype or other clinical factors), made it impossible to detect a statistically significant difference in the effect of individual antifungal agents on tacrolimus clearance. Other co-medications that could potentially influence the pharmacokinetics of tacrolimus are MMP and glucocorticoids. However, as all patients were co-treated with MMP and no patient received glucocorticoids, it was not possible to observe the possible effect of these drugs (due to the absence of a control group). In addition to its direct effect on tacrolimus exposure, immunosuppressive co-medication may also affect the observed outcomes (Pidala et al., 2012). Therefore, it is important to note that our findings may be fully transferable only if the immunosuppressive regimen used is identical.

Tacrolimus is partially distributed in the blood into erythrocytes, which is why it is recommended to measure whole blood levels of this drug. This fact has important implications—when the red blood cell count decreases, tacrolimus levels in total blood may decrease quite significantly, whereas the concentration of the free fraction of the drug may be the same or even increase (which may be associated with a higher risk of toxicity) (Sikma et al., 2020; Sikma et al., 2015). In clinically unstable patients with fluctuating red blood cell counts, some authors, therefore, recommend adjusting the tacrolimus level (and subsequently dosage) according to hematocrit. Nevertheless, no validation studies have yet compared hematocrit-corrected whole blood tacrolimus levels against measured concentrations of unbound drug that could reliably confirm this hypothesis. Patients after allogeneic stem cell transplantation are mostly clinically stable with very predictable hemoglobin (Hb) levels. Around stem cell infusion, their Hb level ranges between 70 and 80 g/L and steadily and uniformly increases to normal levels during first 3 months after transplantation. They do not experience fluid shifts or bleeding complication as they undergo no surgery procedure. We therefore did not consider using hematocrit-adjusted tacrolimus levels in our analyses. On the other hand, it should be noted that the findings of our study are only applicable to comparable patient populations.

**FIGURE 2:**
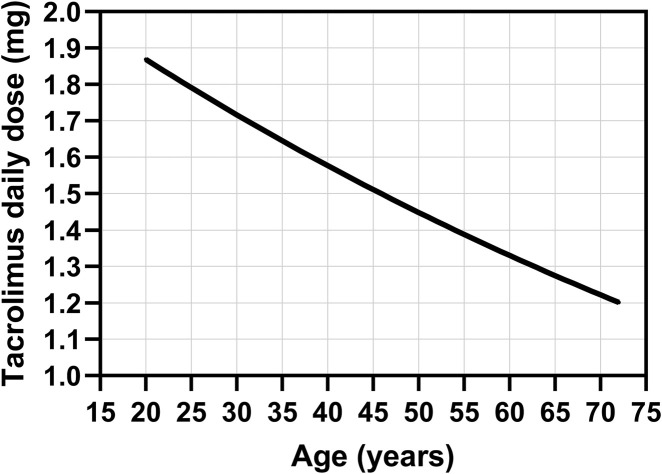
Nomogram for the estimation of the maximum initial daily dose of intravenous tacrolimus in patients undergoing first stem cell transplantation based on the observed association between tacrolimus clearance and age, described in our population model, and considering the emerging AUC_tc_ cutoff value of 279 ng h/mL for tacrolimus exposure.

Based on the observed association between tacrolimus clearance and age, described in our population model, and considering the emerging AUC_tc_ cutoff value of 279 ng h/mL, we propose a nomogram for estimating the maximum initial daily dose of tacrolimus in patients undergoing first stem cell transplantation (Figure 2). This nomogram was constructed based on the equation among dose, clearance, and drug exposition (dose = CL × AUC). This dose should be further reduced by 23% for patients undergoing repeat transplantation. Following this proposed dosage, the cutoff value should not be exceeded. However, due to the relatively high residual variability in tacrolimus pharmacokinetics, it is still necessary to subsequently measure tacrolimus levels and adjust the dose if required, according to TDM principles.

Despite the very uniform protocol for immunosuppression, our study was conducted in a retrospective way, which is its main limitation. Furthermore, identification of cutoff values for tacrolimus exposure using overall survival as an endpoint and then using these cutoff values in the survival analysis of the same dataset may lead to the overestimation of significance of findings. Therefore, the findings of this study need to be validated in a prospectively conducted study. Finally, some studies indicated that CMIA tends to overestimate tacrolimus levels in comparison with the LC/MS method (Becker et al., 2020; Levine et al., 2011). This potential bias may have led to a slight overestimation of the identified tacrolimus thresholds, if applied at a site where tacrolimus is measured using the LC/MS method. On the other hand, to the best of our knowledge, this is the first study to cover such a long period of tacrolimus exposure and to report its time dependence with respect to allogeneic stem cell transplantation. Moreover, it proposes a very practical clinical recommendation for tacrolimus dose optimization.

## 5 Conclusion

We identified a cutoff value for tacrolimus exposure, above which patients’ overall survival is reduced after allogeneic stem cell transplantation. Exceeding the average trough tacrolimus level in the second and third months after transplantation, above 10.8 and 6.7 ng/mL, respectively, reduces the overall survival by approximately three-fold. We also observed that long-term exposure to tacrolimus (a three-month period) was more important for overall survival than exposure in the initial phase (the first month). Other factors influencing survival included the disease risk category, age ≥55 years, and non-myeloablative conditioning. Furthermore, we described a tacrolimus population pharmacokinetic model, in which tacrolimus clearance decreases with age and is reduced by 23% in patients undergoing a repeat transplantation. Based on these observations, we propose a nomogram for determining the maximum initial tacrolimus daily dose in patients treated with allogeneic stem cell transplantation.

## Data Availability

The raw data supporting the conclusions of this article will be made available by the authors, without undue reservation.
