# Supplementary material for: Tacrolimus exposure during the three-month period following allogeneic stem cell transplantation predicts overall survival
Source: Front Pharmacol. 2025 Apr 25;16:1517083. doi: 10.3389/fphar.2025.1517083 (PMC12061681; doi:10.3389/fphar.2025.1517083)
Supplement: Supplementary file 1 [file Supplementaryfile1.docx]

**Supplementary data:**

**Tacrolimus exposure during the three-month period following allogeneic stem cell transplantation predicts overall survival**

**Alzbeta Zavrelova^1^, Katerina Zibridova^1^, Jakub Radocha^1^, Eva Cermakova^2^, Petra Rozsivalova^3^, Pavel Zak^1^, Benjamin Visek^1^, Miriam Lanska^1^, Jana Stevkova^1^, Sara Merdita^4^, Ondrej Slanar^4^, Martin Sima^4*^**

**Affiliations:**

^1^ 4^th^ Department of Internal Medicine – Hematology, University Hospital Hradec Kralove and Faculty of Medicine in Hradec Kralove, Charles University, Hradec Kralove, Czech Republic

^2^ Department of Medical Biophysics, Faculty of Medicine in Hradec Kralove, Charles University, Hradec Kralove, Czech Republic

^3^ Department of Clinical Pharmacy, Hospital Pharmacy, University Hospital Hradec Kralove and Faculty of Pharmacy in Hradec Kralove, Charles University, Hradec Kralove, Czech Republic

^4^ Institute of Pharmacology, First Faculty of Medicine, Charles University and General University Hospital in Prague, Prague, Czech Republic.

**ROC report - identification of cut-off values for tacrolimus exposure for whole period.**

The selected value is marked in bold.

────────────────────────────────────────────────────────────────

**Table Counts**

**────────────────────**

**Cutoff TPs FPs FNs TNs TPR TNR Accur- TPR +**

**Value A B C D (Sens.) (Spec.) PPV acy TNR**

────────────────────────────────────────────────────────────────────────────────────────────────────────────────────────────────

≥ 121.00 35 51 0 0 1.0000 0.0000 0.4070 0.4070 1.0000

≥ 146.00 34 51 1 0 0.9714 0.0000 0.4000 0.3953 0.9714

≥ 149.00 34 50 1 1 0.9714 0.0196 0.4048 0.4070 0.9910

≥ 160.00 34 49 1 2 0.9714 0.0392 0.4096 0.4186 1.0106

≥ 161.00 34 48 1 3 0.9714 0.0588 0.4146 0.4302 1.0303

≥ 174.00 34 47 1 4 0.9714 0.0784 0.4198 0.4419 1.0499

≥ 176.00 34 44 1 7 0.9714 0.1373 0.4359 0.4767 1.1087

≥ 177.00 34 43 1 8 0.9714 0.1569 0.4416 0.4884 1.1283

≥ 179.00 34 42 1 9 0.9714 0.1765 0.4474 0.5000 1.1479

≥ 180.00 34 41 1 10 0.9714 0.1961 0.4533 0.5116 1.1675

≥ 181.00 34 40 1 11 0.9714 0.2157 0.4595 0.5233 1.1871

≥ 182.00 33 39 2 12 0.9429 0.2353 0.4583 0.5233 1.1782

≥ 185.00 32 39 3 12 0.9143 0.2353 0.4507 0.5116 1.1496

≥ 186.00 31 37 4 14 0.8857 0.2745 0.4559 0.5233 1.1602

≥ 188.00 30 34 5 17 0.8571 0.3333 0.4688 0.5465 1.1905

≥ 189.00 30 33 5 18 0.8571 0.3529 0.4762 0.5581 1.2101

≥ 190.00 30 32 5 19 0.8571 0.3725 0.4839 0.5698 1.2297

≥ 191.00 30 31 5 20 0.8571 0.3922 0.4918 0.5814 1.2493

≥ 194.00 29 31 6 20 0.8286 0.3922 0.4833 0.5698 1.2207

≥ 197.00 29 30 6 21 0.8286 0.4118 0.4915 0.5814 1.2403

≥ 199.00 29 29 6 22 0.8286 0.4314 0.5000 0.5930 1.2599

≥ 200.00 29 28 6 23 0.8286 0.4510 0.5088 0.6047 1.2796

≥ 201.00 29 27 6 24 0.8286 0.4706 0.5179 0.6163 1.2992

≥ 202.00 28 27 7 24 0.8000 0.4706 0.5091 0.6047 1.2706

≥ 204.00 28 26 7 25 0.8000 0.4902 0.5185 0.6163 1.2902

≥ 208.00 27 25 8 26 0.7714 0.5098 0.5192 0.6163 1.2812

≥ 209.00 26 25 9 26 0.7429 0.5098 0.5098 0.6047 1.2527

≥ 210.00 26 24 9 27 0.7429 0.5294 0.5200 0.6163 1.2723

≥ 213.00 26 23 9 28 0.7429 0.5490 0.5306 0.6279 1.2919

≥ 214.00 26 22 9 29 0.7429 0.5686 0.5417 0.6395 1.3115

≥ 216.00 25 22 10 29 0.7143 0.5686 0.5319 0.6279 1.2829

≥ 217.00 25 21 10 30 0.7143 0.5882 0.5435 0.6395 1.3025

≥ 218.00 25 20 10 31 0.7143 0.6078 0.5556 0.6512 1.3221

≥ 221.00 24 19 11 32 0.6857 0.6275 0.5581 0.6512 1.3132

**≥ 222.00 24 18 11 33 0.6857 0.6471 0.5714 0.6628 1.3328**

≥ 224.00 23 18 12 33 0.6571 0.6471 0.5610 0.6512 1.3042

≥ 225.00 21 18 14 33 0.6000 0.6471 0.5385 0.6279 1.2471

≥ 227.00 21 17 14 34 0.6000 0.6667 0.5526 0.6395 1.2667

≥ 230.00 20 17 15 34 0.5714 0.6667 0.5405 0.6279 1.2381

≥ 233.00 20 16 15 35 0.5714 0.6863 0.5556 0.6395 1.2577

≥ 238.00 20 15 15 36 0.5714 0.7059 0.5714 0.6512 1.2773

≥ 239.00 20 14 15 37 0.5714 0.7255 0.5882 0.6628 1.2969

≥ 240.00 18 14 17 37 0.5143 0.7255 0.5625 0.6395 1.2398

≥ 242.00 17 13 18 38 0.4857 0.7451 0.5667 0.6395 1.2308

≥ 246.00 16 12 19 39 0.4571 0.7647 0.5714 0.6395 1.2218

≥ 248.00 15 12 20 39 0.4286 0.7647 0.5556 0.6279 1.1933

≥ 253.00 14 11 21 40 0.4000 0.7843 0.5600 0.6279 1.1843

≥ 257.00 13 11 22 40 0.3714 0.7843 0.5417 0.6163 1.1557

≥ 259.00 13 10 22 41 0.3714 0.8039 0.5652 0.6279 1.1754

≥ 262.00 13 9 22 42 0.3714 0.8235 0.5909 0.6395 1.1950

≥ 264.00 13 8 22 43 0.3714 0.8431 0.6190 0.6512 1.2146

≥ 265.00 12 8 23 43 0.3429 0.8431 0.6000 0.6395 1.1860

≥ 274.00 11 8 24 43 0.3143 0.8431 0.5789 0.6279 1.1574

≥ 277.00 10 8 25 43 0.2857 0.8431 0.5556 0.6163 1.1289

≥ 278.00 10 7 25 44 0.2857 0.8627 0.5882 0.6279 1.1485

≥ 279.00 10 6 25 45 0.2857 0.8824 0.6250 0.6395 1.1681

≥ 282.00 9 6 26 45 0.2571 0.8824 0.6000 0.6279 1.1395

≥ 283.00 9 5 26 46 0.2571 0.9020 0.6429 0.6395 1.1591

≥ 285.00 8 5 27 46 0.2286 0.9020 0.6154 0.6279 1.1305

≥ 286.00 8 4 27 47 0.2286 0.9216 0.6667 0.6395 1.1501

≥ 287.00 7 3 28 48 0.2000 0.9412 0.7000 0.6395 1.1412

≥ 288.00 6 2 29 49 0.1714 0.9608 0.7500 0.6395 1.1322

≥ 292.00 5 2 30 49 0.1429 0.9608 0.7143 0.6279 1.1036

≥ 293.00 4 2 31 49 0.1143 0.9608 0.6667 0.6163 1.0751

≥ 302.00 3 2 32 49 0.0857 0.9608 0.6000 0.6047 1.0465

≥ 303.00 2 2 33 49 0.0571 0.9608 0.5000 0.5930 1.0179

≥ 304.00 2 1 33 50 0.0571 0.9804 0.6667 0.6047 1.0375

≥ 328.00 2 0 33 51 0.0571 1.0000 1.0000 0.6163 1.0571

≥ 342.00 1 0 34 51 0.0286 1.0000 1.0000 0.6047 1.0286

────────────────────────────────────────────────────────────────

Definitions:

Cutoff Value Indicates the criterion value range that predicts a positive condition.

A The number of True Positives.

B The number of False Positives.

C The number of False Negatives.

D The number of True Negatives.

TPR The True Positive Rate or Sensitivity = A / (A + C).

TNR The True Negative Rate or Specificity = D / (B + D).

PPV The Positive Predictive Value or Precision = A / (A + B).

Accuracy The Proportion Correctly Classified = (A + D) / (A + B + C + D).

TPR + TNR The Sensitivity + Specificity.

**Area Under Curve Analysis (Empirical Estimation)**

────────────────────────────────────────────────────────────────

Estimated Prevalence = 35 / 86 = 0.4070

Estimated Prevalence is the proportion of the sample with a positive condition of 1. The estimated prevalence

should only be used as a valid estimate of the population prevalence when the entire sample is a random

sample of the population.

────────────────────────────────────────────────────────────────

|  |  |  |  |  |  | 95% Confidence Limits | |
| --- | --- | --- | --- | --- | --- | --- | --- |
| Criterion | Count | AUC | Standard  Error | Z-Value  to Test  AUC > 0.5 | Upper 1-Sided  P-Value | Lower | Upper |
| AUCtc_whole period | 86 | 0.6854 | 0.0584 | 3.176 | 0.0007 | 0.5535 | 0.7838 |

────────────────────────────────────────────────────────────────

Definitions:

Criterion The Criterion Variable containing the scores of the individuals.

Count The number of the individuals used in the analysis.

AUC The area under the ROC curve using the empirical (trapezoidal) approach.

Standard Error The standard error of the AUC estimate.

Z-Value The Z-score for testing the designated hypothesis test.

P-Value The probability level associated with the Z-Value.

Lower and Upper Confidence Limits Form the confidence interval for AUC.

**ROC report - identification of cut-off values for tacrolimus exposure for first month of therapy.**

The selected value is marked in bold.

────────────────────────────────────────────────────────────────

**Table Counts**

**────────────────────**

**Cutoff TPs FPs FNs TNs TPR TNR Accur- TPR +**

**Value A B C D (Sens.) (Spec.) PPV acy TNR**

──────────────────────────────────────────────────────────────────────────────────────────────────────────────────────────────────────

≥ 117.00 35 51 0 0 1.0000 0.0000 0.4070 0.4070 1.0000

≥ 120.00 34 51 1 0 0.9714 0.0000 0.4000 0.3953 0.9714

≥ 164.00 33 51 2 0 0.9429 0.0000 0.3929 0.3837 0.9429

≥ 165.00 33 50 2 1 0.9429 0.0196 0.3976 0.3953 0.9625

≥ 167.00 33 49 2 2 0.9429 0.0392 0.4024 0.4070 0.9821

≥ 168.00 33 48 2 3 0.9429 0.0588 0.4074 0.4186 1.0017

≥ 171.00 32 48 3 3 0.9143 0.0588 0.4000 0.4070 0.9731

≥ 173.00 32 47 3 4 0.9143 0.0784 0.4051 0.4186 0.9927

≥ 183.00 32 46 3 5 0.9143 0.0980 0.4103 0.4302 1.0123

≥ 186.00 32 45 3 6 0.9143 0.1176 0.4156 0.4419 1.0319

≥ 187.00 31 45 4 6 0.8857 0.1176 0.4079 0.4302 1.0034

≥ 194.00 31 44 4 7 0.8857 0.1373 0.4133 0.4419 1.0230

≥ 210.00 31 43 4 8 0.8857 0.1569 0.4189 0.4535 1.0426

≥ 211.00 31 42 4 9 0.8857 0.1765 0.4247 0.4651 1.0622

≥ 214.00 31 40 4 11 0.8857 0.2157 0.4366 0.4884 1.1014

≥ 215.00 31 38 4 13 0.8857 0.2549 0.4493 0.5116 1.1406

≥ 224.00 31 37 4 14 0.8857 0.2745 0.4559 0.5233 1.1602

≥ 226.00 30 37 5 14 0.8571 0.2745 0.4478 0.5116 1.1317

≥ 229.00 30 36 5 15 0.8571 0.2941 0.4545 0.5233 1.1513

≥ 232.00 30 35 5 16 0.8571 0.3137 0.4615 0.5349 1.1709

≥ 235.00 29 35 6 16 0.8286 0.3137 0.4531 0.5233 1.1423

≥ 239.00 28 35 7 16 0.8000 0.3137 0.4444 0.5116 1.1137

≥ 241.00 27 35 8 16 0.7714 0.3137 0.4355 0.5000 1.0852

≥ 244.00 27 34 8 17 0.7714 0.3333 0.4426 0.5116 1.1048

≥ 252.00 27 33 8 18 0.7714 0.3529 0.4500 0.5233 1.1244

≥ 253.00 27 32 8 19 0.7714 0.3725 0.4576 0.5349 1.1440

≥ 257.00 26 32 9 19 0.7429 0.3725 0.4483 0.5233 1.1154

≥ 261.00 26 31 9 20 0.7429 0.3922 0.4561 0.5349 1.1350

≥ 262.00 26 27 9 24 0.7429 0.4706 0.4906 0.5814 1.2134

≥ 266.00 26 26 9 25 0.7429 0.4902 0.5000 0.5930 1.2331

≥ 267.00 25 26 10 25 0.7143 0.4902 0.4902 0.5814 1.2045

≥ 274.00 24 26 11 25 0.6857 0.4902 0.4800 0.5698 1.1759

≥ 277.00 24 25 11 26 0.6857 0.5098 0.4898 0.5814 1.1955

**≥ 279.00 24 24 11 27 0.6857 0.5294 0.5000 0.5930 1.2151**

≥ 281.00 23 24 12 27 0.6571 0.5294 0.4894 0.5814 1.1866

≥ 282.00 23 23 12 28 0.6571 0.5490 0.5000 0.5930 1.2062

≥ 283.00 21 23 14 28 0.6000 0.5490 0.4773 0.5698 1.1490

≥ 284.00 21 22 14 29 0.6000 0.5686 0.4884 0.5814 1.1686

≥ 288.00 21 21 14 30 0.6000 0.5882 0.5000 0.5930 1.1882

≥ 289.00 21 20 14 31 0.6000 0.6078 0.5122 0.6047 1.2078

≥ 292.00 20 20 15 31 0.5714 0.6078 0.5000 0.5930 1.1793

≥ 293.00 20 19 15 32 0.5714 0.6275 0.5128 0.6047 1.1989

≥ 296.00 19 18 16 33 0.5429 0.6471 0.5135 0.6047 1.1899

≥ 298.00 18 18 17 33 0.5143 0.6471 0.5000 0.5930 1.1613

≥ 299.00 17 18 18 33 0.4857 0.6471 0.4857 0.5814 1.1328

≥ 300.00 16 18 19 33 0.4571 0.6471 0.4706 0.5698 1.1042

≥ 302.00 16 17 19 34 0.4571 0.6667 0.4848 0.5814 1.1238

≥ 303.00 16 16 19 35 0.4571 0.6863 0.5000 0.5930 1.1434

≥ 306.00 16 15 19 36 0.4571 0.7059 0.5161 0.6047 1.1630

≥ 311.00 14 15 21 36 0.4000 0.7059 0.4828 0.5814 1.1059

≥ 312.00 13 15 22 36 0.3714 0.7059 0.4643 0.5698 1.0773

≥ 313.00 12 15 23 36 0.3429 0.7059 0.4444 0.5581 1.0487

≥ 315.00 12 14 23 37 0.3429 0.7255 0.4615 0.5698 1.0683

≥ 320.00 11 14 24 37 0.3143 0.7255 0.4400 0.5581 1.0398

≥ 322.00 11 13 24 38 0.3143 0.7451 0.4583 0.5698 1.0594

≥ 323.00 11 11 24 40 0.3143 0.7843 0.5000 0.5930 1.0986

≥ 326.00 10 11 25 40 0.2857 0.7843 0.4762 0.5814 1.0700

≥ 327.00 10 10 25 41 0.2857 0.8039 0.5000 0.5930 1.0896

≥ 329.00 9 10 26 41 0.2571 0.8039 0.4737 0.5814 1.0611

≥ 330.00 8 10 27 41 0.2286 0.8039 0.4444 0.5698 1.0325

≥ 332.00 8 9 27 42 0.2286 0.8235 0.4706 0.5814 1.0521

≥ 334.00 8 8 27 43 0.2286 0.8431 0.5000 0.5930 1.0717

≥ 336.00 8 7 27 44 0.2286 0.8627 0.5333 0.6047 1.0913

≥ 340.00 8 6 27 45 0.2286 0.8824 0.5714 0.6163 1.1109

≥ 342.00 8 5 27 46 0.2286 0.9020 0.6154 0.6279 1.1305

≥ 345.00 7 5 28 46 0.2000 0.9020 0.5833 0.6163 1.1020

≥ 353.00 7 4 28 47 0.2000 0.9216 0.6364 0.6279 1.1216

≥ 364.00 7 3 28 48 0.2000 0.9412 0.7000 0.6395 1.1412

≥ 367.00 6 3 29 48 0.1714 0.9412 0.6667 0.6279 1.1126

≥ 385.00 5 3 30 48 0.1429 0.9412 0.6250 0.6163 1.0840

≥ 392.00 5 2 30 49 0.1429 0.9608 0.7143 0.6279 1.1036

≥ 403.00 5 1 30 50 0.1429 0.9804 0.8333 0.6395 1.1232

≥ 421.00 5 0 30 51 0.1429 1.0000 1.0000 0.6512 1.1429

≥ 430.00 4 0 31 51 0.1143 1.0000 1.0000 0.6395 1.1143

≥ 431.00 3 0 32 51 0.0857 1.0000 1.0000 0.6279 1.0857

≥ 464.00 2 0 33 51 0.0571 1.0000 1.0000 0.6163 1.0571

≥ 503.00 1 0 34 51 0.0286 1.0000 1.0000 0.6047 1.0286

────────────────────────────────────────────────────────────────

Definitions:

Cutoff Value Indicates the criterion value range that predicts a positive condition.

A The number of True Positives.

B The number of False Positives.

C The number of False Negatives.

D The number of True Negatives.

TPR The True Positive Rate or Sensitivity = A / (A + C).

TNR The True Negative Rate or Specificity = D / (B + D).

PPV The Positive Predictive Value or Precision = A / (A + B).

Accuracy The Proportion Correctly Classified = (A + D) / (A + B + C + D).

TPR + TNR The Sensitivity + Specificity.

**Area Under Curve Analysis (Empirical Estimation)**

────────────────────────────────────────────────────────────────

Estimated Prevalence = 35 / 86 = 0.4070

Estimated Prevalence is the proportion of the sample with a positive condition of 1. The estimated prevalence

should only be used as a valid estimate of the population prevalence when the entire sample is a random

sample of the population.

────────────────────────────────────────────────────────────────

|  |  |  |  |  |  | 95% Confidence Limits | |
| --- | --- | --- | --- | --- | --- | --- | --- |
| Criterion | Count | AUC | Standard  Error | Z-Value  to Test  AUC > 0.5 | Upper 1-Sided  P-Value | Lower | Upper |
| AUCtc_1st month | 86 | 0.6081 | 0.0632 | 1.711 | 0.0435 | 0.4695 | 0.7175 |

────────────────────────────────────────────────────────────────

Definitions:

Criterion The Criterion Variable containing the scores of the individuals.

Count The number of the individuals used in the analysis.

AUC The area under the ROC curve using the empirical (trapezoidal) approach.

Standard Error The standard error of the AUC estimate.

Z-Value The Z-score for testing the designated hypothesis test.

P-Value The probability level associated with the Z-Value.

Lower and Upper Confidence Limits Form the confidence interval for AUC.

**ROC report - identification of cut-off values for tacrolimus exposure for second month of therapy.**

The selected value is marked in bold.

────────────────────────────────────────────────────────────────

**Table Counts**

**────────────────────**

**Cutoff TPs FPs FNs TNs TPR TNR Accur- TPR +**

**Value A B C D (Sens.) (Spec.) PPV acy TNR**

────────────────────────────────────────────────────────────────────────────────────────────────────────────────────────────────

≥ 105.80 31 51 0 0 1.0000 0.0000 0.3780 0.3780 1.0000

≥ 119.30 31 50 0 1 1.0000 0.0196 0.3827 0.3902 1.0196

≥ 126.50 31 49 0 2 1.0000 0.0392 0.3875 0.4024 1.0392

≥ 133.30 30 49 1 2 0.9677 0.0392 0.3797 0.3902 1.0070

≥ 138.70 30 48 1 3 0.9677 0.0588 0.3846 0.4024 1.0266

≥ 143.50 30 47 1 4 0.9677 0.0784 0.3896 0.4146 1.0462

≥ 149.80 30 46 1 5 0.9677 0.0980 0.3947 0.4268 1.0658

≥ 151.50 30 45 1 6 0.9677 0.1176 0.4000 0.4390 1.0854

≥ 152.40 29 45 2 6 0.9355 0.1176 0.3919 0.4268 1.0531

≥ 152.50 29 44 2 7 0.9355 0.1373 0.3973 0.4390 1.0727

≥ 154.50 29 43 2 8 0.9355 0.1569 0.4028 0.4512 1.0923

≥ 156.80 29 42 2 9 0.9355 0.1765 0.4085 0.4634 1.1120

≥ 158.90 29 41 2 10 0.9355 0.1961 0.4143 0.4756 1.1316

≥ 171.10 29 40 2 11 0.9355 0.2157 0.4203 0.4878 1.1512

≥ 172.10 28 40 3 11 0.9032 0.2157 0.4118 0.4756 1.1189

≥ 178.80 27 40 4 11 0.8710 0.2157 0.4030 0.4634 1.0867

≥ 178.90 27 39 4 12 0.8710 0.2353 0.4091 0.4756 1.1063

≥ 181.20 27 38 4 13 0.8710 0.2549 0.4154 0.4878 1.1259

≥ 182.20 26 38 5 13 0.8387 0.2549 0.4063 0.4756 1.0936

≥ 182.30 25 38 6 13 0.8065 0.2549 0.3968 0.4634 1.0614

≥ 185.60 25 37 6 14 0.8065 0.2745 0.4032 0.4756 1.0810

≥ 189.90 24 37 7 14 0.7742 0.2745 0.3934 0.4634 1.0487

≥ 194.60 23 37 8 14 0.7419 0.2745 0.3833 0.4512 1.0164

≥ 196.20 23 35 8 16 0.7419 0.3137 0.3966 0.4756 1.0557

≥ 202.20 23 34 8 17 0.7419 0.3333 0.4035 0.4878 1.0753

≥ 202.40 22 34 9 17 0.7097 0.3333 0.3929 0.4756 1.0430

≥ 206.10 22 33 9 18 0.7097 0.3529 0.4000 0.4878 1.0626

≥ 207.30 22 32 9 19 0.7097 0.3725 0.4074 0.5000 1.0822

≥ 210.50 22 31 9 20 0.7097 0.3922 0.4151 0.5122 1.1018

≥ 211.10 22 30 9 21 0.7097 0.4118 0.4231 0.5244 1.1214

≥ 213.10 22 29 9 22 0.7097 0.4314 0.4314 0.5366 1.1410

≥ 218.70 22 28 9 23 0.7097 0.4510 0.4400 0.5488 1.1607

≥ 224.20 21 28 10 23 0.6774 0.4510 0.4286 0.5366 1.1284

≥ 230.10 20 28 11 23 0.6452 0.4510 0.4167 0.5244 1.0961

≥ 231.10 20 27 11 24 0.6452 0.4706 0.4255 0.5366 1.1157

≥ 235.20 20 26 11 25 0.6452 0.4902 0.4348 0.5488 1.1354

≥ 235.40 20 25 11 26 0.6452 0.5098 0.4444 0.5610 1.1550

≥ 237.60 19 25 12 26 0.6129 0.5098 0.4318 0.5488 1.1227

≥ 237.90 19 24 12 27 0.6129 0.5294 0.4419 0.5610 1.1423

≥ 239.50 19 23 12 28 0.6129 0.5490 0.4524 0.5732 1.1619

≥ 243.90 19 22 12 29 0.6129 0.5686 0.4634 0.5854 1.1815

≥ 244.60 19 21 12 30 0.6129 0.5882 0.4750 0.5976 1.2011

≥ 248.60 19 20 12 31 0.6129 0.6078 0.4872 0.6098 1.2207

≥ 249.00 19 19 12 32 0.6129 0.6275 0.5000 0.6220 1.2404

≥ 249.10 18 19 13 32 0.5806 0.6275 0.4865 0.6098 1.2081

≥ 255.20 18 18 13 33 0.5806 0.6471 0.5000 0.6220 1.2277

≥ 255.30 17 18 14 33 0.5484 0.6471 0.4857 0.6098 1.1954

≥ 255.90 17 17 14 34 0.5484 0.6667 0.5000 0.6220 1.2151

≥ 256.80 17 16 14 35 0.5484 0.6863 0.5152 0.6341 1.2347

**≥ 258.40 17 15 14 36 0.5484 0.7059 0.5313 0.6463 1.2543**

≥ 262.20 16 15 15 36 0.5161 0.7059 0.5161 0.6341 1.2220

≥ 267.30 15 15 16 36 0.4839 0.7059 0.5000 0.6220 1.1898

≥ 268.50 14 15 17 36 0.4516 0.7059 0.4828 0.6098 1.1575

≥ 269.30 14 14 17 37 0.4516 0.7255 0.5000 0.6220 1.1771

≥ 271.40 13 14 18 37 0.4194 0.7255 0.4815 0.6098 1.1448

≥ 280.60 13 13 18 38 0.4194 0.7451 0.5000 0.6220 1.1645

≥ 282.30 12 13 19 38 0.3871 0.7451 0.4800 0.6098 1.1322

≥ 282.80 11 13 20 38 0.3548 0.7451 0.4583 0.5976 1.0999

≥ 286.20 10 13 21 38 0.3226 0.7451 0.4348 0.5854 1.0677

≥ 289.90 9 13 22 38 0.2903 0.7451 0.4091 0.5732 1.0354

≥ 290.30 8 13 23 38 0.2581 0.7451 0.3810 0.5610 1.0032

≥ 292.10 7 13 24 38 0.2258 0.7451 0.3500 0.5488 0.9709

≥ 292.80 6 13 25 38 0.1935 0.7451 0.3158 0.5366 0.9386

≥ 294.30 6 12 25 39 0.1935 0.7647 0.3333 0.5488 0.9583

≥ 295.70 5 12 26 39 0.1613 0.7647 0.2941 0.5366 0.9260

≥ 296.10 5 11 26 40 0.1613 0.7843 0.3125 0.5488 0.9456

≥ 302.60 5 10 26 41 0.1613 0.8039 0.3333 0.5610 0.9652

≥ 308.10 5 9 26 42 0.1613 0.8235 0.3571 0.5732 0.9848

≥ 310.00 5 8 26 43 0.1613 0.8431 0.3846 0.5854 1.0044

≥ 315.60 5 7 26 44 0.1613 0.8627 0.4167 0.5976 1.0240

≥ 321.30 4 7 27 44 0.1290 0.8627 0.3636 0.5854 0.9918

≥ 324.50 4 6 27 45 0.1290 0.8824 0.4000 0.5976 1.0114

≥ 325.90 4 5 27 46 0.1290 0.9020 0.4444 0.6098 1.0310

≥ 326.30 4 4 27 47 0.1290 0.9216 0.5000 0.6220 1.0506

≥ 331.30 3 4 28 47 0.0968 0.9216 0.4286 0.6098 1.0183

≥ 372.50 3 3 28 48 0.0968 0.9412 0.5000 0.6220 1.0380

≥ 391.60 3 2 28 49 0.0968 0.9608 0.6000 0.6341 1.0576

≥ 401.00 2 2 29 49 0.0645 0.9608 0.5000 0.6220 1.0253

≥ 412.80 2 1 29 50 0.0645 0.9804 0.6667 0.6341 1.0449

≥ 442.90 2 0 29 51 0.0645 1.0000 1.0000 0.6463 1.0645

≥ 539.70 1 0 30 51 0.0323 1.0000 1.0000 0.6341 1.0323

────────────────────────────────────────────────────────────────

Definitions:

Cutoff Value Indicates the criterion value range that predicts a positive condition.

A The number of True Positives.

B The number of False Positives.

C The number of False Negatives.

D The number of True Negatives.

TPR The True Positive Rate or Sensitivity = A / (A + C).

TNR The True Negative Rate or Specificity = D / (B + D).

PPV The Positive Predictive Value or Precision = A / (A + B).

Accuracy The Proportion Correctly Classified = (A + D) / (A + B + C + D).

TPR + TNR The Sensitivity + Specificity.

**Area Under Curve Analysis (Empirical Estimation)**

────────────────────────────────────────────────────────────────

Estimated Prevalence = 31 / 82 = 0.3780

Estimated Prevalence is the proportion of the sample with a positive condition of 1. The estimated prevalence

should only be used as a valid estimate of the population prevalence when the entire sample is a random

sample of the population.

────────────────────────────────────────────────────────────────

|  |  |  |  |  |  | 95% Confidence Limits | |
| --- | --- | --- | --- | --- | --- | --- | --- |
| Criterion | Count | AUC | Standard  Error | Z-Value  to Test  AUC > 0.5 | Upper 1-Sided  P-Value | Lower | Upper |
| AUCtc_2nd month | 82 | 0.5882 | 0.0653 | 1.351 | 0.0884 | 0.4456 | 0.7018 |

────────────────────────────────────────────────────────────────

Definitions:

Criterion The Criterion Variable containing the scores of the individuals.

Count The number of the individuals used in the analysis.

AUC The area under the ROC curve using the empirical (trapezoidal) approach.

Standard Error The standard error of the AUC estimate.

Z-Value The Z-score for testing the designated hypothesis test.

P-Value The probability level associated with the Z-Value.

Lower and Upper Confidence Limits Form the confidence interval for AUC.

**ROC report - identification of cut-off values for tacrolimus exposure for third month of therapy.**

The selected value is marked in bold.

────────────────────────────────────────────────────────────────

**Table Counts**

**────────────────────**

**Cutoff TPs FPs FNs TNs TPR TNR Accur- TPR +**

**Value A B C D (Sens.) (Spec.) PPV acy TNR**

──────────────────────────────────────────────────────────────────────────────────────────────────────────────────────────────────────

≥ 63.00 31 51 0 0 1.0000 0.0000 0.3780 0.3780 1.0000

≥ 65.00 31 50 0 1 1.0000 0.0196 0.3827 0.3902 1.0196

≥ 75.00 31 49 0 2 1.0000 0.0392 0.3875 0.4024 1.0392

≥ 77.00 31 48 0 3 1.0000 0.0588 0.3924 0.4146 1.0588

≥ 83.00 30 48 1 3 0.9677 0.0588 0.3846 0.4024 1.0266

≥ 94.00 30 47 1 4 0.9677 0.0784 0.3896 0.4146 1.0462

≥ 97.00 29 47 2 4 0.9355 0.0784 0.3816 0.4024 1.0139

≥ 102.00 29 46 2 5 0.9355 0.0980 0.3867 0.4146 1.0335

≥ 104.00 28 46 3 5 0.9032 0.0980 0.3784 0.4024 1.0013

≥ 105.00 27 45 4 6 0.8710 0.1176 0.3750 0.4024 0.9886

≥ 108.00 27 44 4 7 0.8710 0.1373 0.3803 0.4146 1.0082

≥ 109.00 27 42 4 9 0.8710 0.1765 0.3913 0.4390 1.0474

≥ 116.00 27 41 4 10 0.8710 0.1961 0.3971 0.4512 1.0670

≥ 118.00 27 40 4 11 0.8710 0.2157 0.4030 0.4634 1.0867

≥ 122.00 27 39 4 12 0.8710 0.2353 0.4091 0.4756 1.1063

≥ 123.00 27 38 4 13 0.8710 0.2549 0.4154 0.4878 1.1259

≥ 125.00 26 38 5 13 0.8387 0.2549 0.4063 0.4756 1.0936

≥ 126.00 25 37 6 14 0.8065 0.2745 0.4032 0.4756 1.0810

≥ 128.00 25 36 6 15 0.8065 0.2941 0.4098 0.4878 1.1006

≥ 131.00 25 35 6 16 0.8065 0.3137 0.4167 0.5000 1.1202

≥ 132.00 25 34 6 17 0.8065 0.3333 0.4237 0.5122 1.1398

≥ 133.00 25 33 6 18 0.8065 0.3529 0.4310 0.5244 1.1594

≥ 137.00 25 31 6 20 0.8065 0.3922 0.4464 0.5488 1.1986

≥ 138.00 24 31 7 20 0.7742 0.3922 0.4364 0.5366 1.1664

≥ 147.00 24 30 7 21 0.7742 0.4118 0.4444 0.5488 1.1860

≥ 148.00 24 29 7 22 0.7742 0.4314 0.4528 0.5610 1.2056

≥ 152.00 24 28 7 23 0.7742 0.4510 0.4615 0.5732 1.2252

≥ 153.00 24 27 7 24 0.7742 0.4706 0.4706 0.5854 1.2448

≥ 155.00 24 26 7 25 0.7742 0.4902 0.4800 0.5976 1.2644

≥ 158.00 24 25 7 26 0.7742 0.5098 0.4898 0.6098 1.2840

≥ 159.00 24 24 7 27 0.7742 0.5294 0.5000 0.6220 1.3036

**≥ 160.00 24 23 7 28 0.7742 0.5490 0.5106 0.6341 1.3232**

≥ 162.00 23 22 8 29 0.7419 0.5686 0.5111 0.6341 1.3106

≥ 169.00 22 22 9 29 0.7097 0.5686 0.5000 0.6220 1.2783

≥ 172.00 22 21 9 30 0.7097 0.5882 0.5116 0.6341 1.2979

≥ 174.00 21 21 10 30 0.6774 0.5882 0.5000 0.6220 1.2657

≥ 175.00 20 21 11 30 0.6452 0.5882 0.4878 0.6098 1.2334

≥ 178.00 20 20 11 31 0.6452 0.6078 0.5000 0.6220 1.2530

≥ 184.00 20 19 11 32 0.6452 0.6275 0.5128 0.6341 1.2726

≥ 186.00 20 18 11 33 0.6452 0.6471 0.5263 0.6463 1.2922

≥ 190.00 19 18 12 33 0.6129 0.6471 0.5135 0.6341 1.2600

≥ 192.00 19 17 12 34 0.6129 0.6667 0.5278 0.6463 1.2796

≥ 194.00 18 17 13 34 0.5806 0.6667 0.5143 0.6341 1.2473

≥ 195.00 17 17 14 34 0.5484 0.6667 0.5000 0.6220 1.2151

≥ 197.00 17 16 14 35 0.5484 0.6863 0.5152 0.6341 1.2347

≥ 199.00 17 15 14 36 0.5484 0.7059 0.5313 0.6463 1.2543

≥ 202.00 17 14 14 37 0.5484 0.7255 0.5484 0.6585 1.2739

≥ 205.00 16 13 15 38 0.5161 0.7451 0.5517 0.6585 1.2612

≥ 206.00 16 12 15 39 0.5161 0.7647 0.5714 0.6707 1.2808

≥ 215.00 16 11 15 40 0.5161 0.7843 0.5926 0.6829 1.3004

≥ 217.00 15 10 16 41 0.4839 0.8039 0.6000 0.6829 1.2878

≥ 219.00 14 10 17 41 0.4516 0.8039 0.5833 0.6707 1.2555

≥ 220.00 14 9 17 42 0.4516 0.8235 0.6087 0.6829 1.2751

≥ 226.00 14 7 17 44 0.4516 0.8627 0.6667 0.7073 1.3144

≥ 229.00 13 7 18 44 0.4194 0.8627 0.6500 0.6951 1.2821

≥ 233.00 13 6 18 45 0.4194 0.8824 0.6842 0.7073 1.3017

≥ 235.00 12 6 19 45 0.3871 0.8824 0.6667 0.6951 1.2694

≥ 238.00 10 6 21 45 0.3226 0.8824 0.6250 0.6707 1.2049

≥ 242.00 9 5 22 46 0.2903 0.9020 0.6429 0.6707 1.1923

≥ 244.00 8 5 23 46 0.2581 0.9020 0.6154 0.6585 1.1600

≥ 254.00 7 5 24 46 0.2258 0.9020 0.5833 0.6463 1.1278

≥ 274.00 6 5 25 46 0.1935 0.9020 0.5455 0.6341 1.0955

≥ 283.00 6 4 25 47 0.1935 0.9216 0.6000 0.6463 1.1151

≥ 294.00 5 4 26 47 0.1613 0.9216 0.5556 0.6341 1.0829

≥ 297.00 5 3 26 48 0.1613 0.9412 0.6250 0.6463 1.1025

≥ 302.00 4 3 27 48 0.1290 0.9412 0.5714 0.6341 1.0702

≥ 311.00 4 2 27 49 0.1290 0.9608 0.6667 0.6463 1.0898

≥ 312.00 4 1 27 50 0.1290 0.9804 0.8000 0.6585 1.1094

≥ 315.00 3 1 28 50 0.0968 0.9804 0.7500 0.6463 1.0772

≥ 329.00 2 1 29 50 0.0645 0.9804 0.6667 0.6341 1.0449

≥ 340.00 1 1 30 50 0.0323 0.9804 0.5000 0.6220 1.0127

≥ 470.00 1 0 30 51 0.0323 1.0000 1.0000 0.6341 1.0323

────────────────────────────────────────────────────────────────

Definitions:

Cutoff Value Indicates the criterion value range that predicts a positive condition.

A The number of True Positives.

B The number of False Positives.

C The number of False Negatives.

D The number of True Negatives.

TPR The True Positive Rate or Sensitivity = A / (A + C).

TNR The True Negative Rate or Specificity = D / (B + D).

PPV The Positive Predictive Value or Precision = A / (A + B).

Accuracy The Proportion Correctly Classified = (A + D) / (A + B + C + D).

TPR + TNR The Sensitivity + Specificity.

**Area Under Curve Analysis (Empirical Estimation)**

────────────────────────────────────────────────────────────────

Estimated Prevalence = 31 / 82 = 0.3780

Estimated Prevalence is the proportion of the sample with a positive condition of 1. The estimated prevalence

should only be used as a valid estimate of the population prevalence when the entire sample is a random

sample of the population.

────────────────────────────────────────────────────────────────

|  |  |  |  |  |  | 95% Confidence Limits | |
| --- | --- | --- | --- | --- | --- | --- | --- |
| Criterion | Count | AUC | Standard  Error | Z-Value  to Test  AUC > 0.5 | Upper 1-Sided  P-Value | Lower | Upper |
| AUCtc_3rd month | 82 | 0.6705 | 0.0645 | 2.643 | 0.0041 | 0.5241 | 0.7784 |

────────────────────────────────────────────────────────────────

Definitions:

Criterion The Criterion Variable containing the scores of the individuals.

Count The number of the individuals used in the analysis.

AUC The area under the ROC curve using the empirical (trapezoidal) approach.

Standard Error The standard error of the AUC estimate.

Z-Value The Z-score for testing the designated hypothesis test.

P-Value The probability level associated with the Z-Value.

Lower and Upper Confidence Limits Form the confidence interval for AUC.
